# Resistance Training in Type II Diabetes Mellitus: Impact on Areas of Metabolic Dysfunction in Skeletal Muscle and Potential Impact on Bone

**DOI:** 10.1155/2012/268197

**Published:** 2012-02-06

**Authors:** Richard J. Wood, Elizabeth C. O'Neill

**Affiliations:** Department of Exercise Science & Sport Studies, Springfield College, 263 Alden St. Athletic Training/Exercise Science Complex, Springfield, MA 01109, USA

## Abstract

The prevalence of Type II Diabetes mellitus (T2DM) is increasing rapidly and will continue to be a major healthcare expenditure burden. As such, identification of effective lifestyle treatments is paramount. Skeletal muscle and bone display metabolic and functional disruption in T2DM. Skeletal muscle in T2DM is characterized by insulin resistance, impaired glycogen synthesis, impairments in mitochondria, and lipid accumulation. Bone quality in T2DM is decreased, potentially due to the effects of advanced glycation endproducts on collagen, impaired osteoblast activity, and lipid accumulation. Although exercise is widely recognized as an important component of treatment for T2DM, the focus has largely been on aerobic exercise. Emerging research suggests that resistance training (strength training) may impose potent and unique benefits in T2DM. The purpose of this review is to examine the role of resistance training in treating the dysfunction in skeletal muscle and the potential role for resistance training in treating the associated dysfunction in bone.

## 1. Introduction

The rapid increase in incidence of Type 2 Diabetes Mellitus (T2DM) underscores the importance of identifying effective treatment strategies. Between 2000 and 2030, the number of people with T2DM worldwide is expected to rise from 171 million to 366 million, and this increase is expected even if current levels of obesity remain constant [1].

In adults, T2DM accounts for 90–95% of all diagnosed cases of diabetes. Insulin resistance is usually an early characteristic of T2DM. The chronic need for elevated insulin secretion can lead an inability for pancreatic production to meet needs and ultimately to *β*-cell failure.

Several comorbidities to T2DM exist including cardiovascular disease, retinopathy, nephropathy, hypertension, and amputation. According to the National Diabetes Fact Sheet, published by the Centers for Disease Control, the total direct cost for diagnosed cases of diabetes in the United States in 2007 was $174 billion, which is startling given that the total number of people with diabetes increases by 37% when including undiagnosed cases [2].

Though the etiology of T2DM is unclear and likely multifactorial, a considerable body of evidence has identified dysfunction in both skeletal muscle and bone in T2DM. People with T2DM display insulin resistance in skeletal muscle [3], characterized by buildup of intramuscular triglyceride [4, 5], and impaired mitochondrial function [6], and this dysfunction has been implicated in the etiology of T2DM. Bone also appears to be affected by T2DM. Fracture rates are 29% higher for diabetic populations as compared to those without diabetes. When examined according to specific fracture sites, risk of fracture in people with T2DM can be twice as high as compared to people without T2DM [7]. Although conflicting data exist, some studies suggest that bone mineral density is actually higher in people with T2DM; yet the quality of the bone is reduced, potentially as a consequence of reduced osteoblast cell growth [8, 9] or excessive lipid accumulation within bone [8].

Over the coming decades, the identification of effective treatment strategies will be key to minimizing the incredible burden of T2DM on healthcare costs. Though often a critical part of the treatment algorithm, drug therapy is expensive and can be accompanied by serious side effects [10]. The identification of effective lifestyle modification strategies is critically important for practitioners and researchers. The inclusion of exercise in lifestyle management provides benefits beyond dietary modification alone, making exercise an important part of lifestyle therapy. Though an abundance of research involving exercise in the treatment of T2DM has focused on aerobic training, a growing body of evidence supports the importance of resistance training as a part of lifestyle therapy. Further, in people with T2DM, resistance training has been shown to positively impact glycemic control [11], adiposity [12], and lipids [13], in many cases to a similar degree to aerobic training [14, 15]. The purpose of this review is to (1) examine the impact of resistance training on the dysfunction in skeletal muscle and (2) hypothesize about the potential role of resistance training on bone in people with T2DM.

## 2. Musculoskeletal Dysfunction in T2DM

### 2.1. Dysfunction in Skeletal Muscle in T2DM (This Section Highlights the Effects of Diabetes on Skeletal Muscle)

#### 2.1.1. Insulin Resistance

Insulin resistance is the reduced response of a target tissue (including skeletal muscle, adipose tissue, etc.) to insulin as compared to a healthy control. In essence, insulin is ineffective despite elevated concentrations. Skeletal muscle is the primary site for insulin-mediated glucose uptake in the postprandial state. Insulin binds the receptor in skeletal muscle, which causes the phosphorylation of tyrosine molecules on the insulin receptor. This causes the insulin receptor substrate-1 (IRS-1) to move to the cell membrane and become phosphorylated on adjacent tyrosine molecules. Next, phosphatidylinositol-3 kinase (PI-3 kinase) is activated, causing the downstream activation of Akt (also called protein kinase B) and the phosphorylation of Akt substrate 160 (AS160), which ultimately facilitates the translocation of GLUT4 to the sarcolemma. GLUT4 is responsible for the transport of glucose into skeletal muscle cells (TANIGUCHI CM 2006 7). Skeletal muscle in people with T2DM typically displays some degree of insulin resistance, characterized by a disruption in the signaling cascade described previously, specifically defective tyrosine phosphorylation of IRS-1 and defects in PI-3 kinase and Akt activation (KROOK A 2000 49; CUSI K 2000 105). DeFronzo et al. [16] found that insulin-stimulated leg glucose uptake is reduced by 50% in people with T2DM.

Insulin resistance in skeletal muscle is considered by some to be the primary defect in T2DM [3] given that it occurs decades before *β*-cell failure [17]. Using a cross-sectional design, Perseghin et al. [18] found that lean, normoglycemic, sedentary offspring of patients with T2DM had significantly lower (~27%) skeletal muscle insulin sensitivity as compared to healthy control subjects. The offspring of patients with T2DM also displayed significantly higher (~31%) fasting insulin. Jallut et al. [19] found that the progression from normal glucose tolerance to impaired glucose tolerance is characterized by a substantial reduction in insulin sensitivity, but serum glucose response to an oral glucose tolerance test was only modestly impacted. Together, these studies and many others [20–22] indicate that skeletal muscle insulin resistance is an early defect in T2DM.

Though cause and effect has not been clearly elucidated, a number of areas of dysfunction within skeletal muscle are related to the disruptions caused to the insulin signaling cascade and ultimately to the degree of insulin resistance. These include impairments in glycogen synthesis, impaired mitochondrial function, and lipid accumulation around and within muscle.

#### 2.1.2. Impaired Glycogen Synthesis

After consumption of carbohydrate, consequent blood glucose elevations cause the secretion of insulin, which stimulates glucose uptake by the liver and skeletal muscle. Under euglycemic hyperinsulinemic conditions, approximately 80% of glucose uptake occurs in skeletal muscle [23]. Once taken up by the cell, glucose can either be oxidized to carbon dioxide and water or converted to glycogen [23], the latter being regulated by glycogen synthase. The impairment of glycogen synthase activity is thought to be one of the earliest defects in skeletal muscle seen in T2DM [24]. Using nuclear magnetic resonance, Perseghin et al. [18] studied glycogen synthesis in the offspring of two parents with T2DM with normal glucose tolerance and found that reductions in glycogen synthesis could almost entirely account for reductions in insulin-stimulated glucose uptake in skeletal muscle. Using similar techniques, Shulman et al. [25] found that glycogen synthesis rates were approximately 57% lower in patients with T2DM as compared to healthy controls. However, using muscle biopsy and subsequent quantitative histology, He and Kelley [26] found that muscle glycogen content was similar between lean non-diabetic, obese non-diabetic, and obese diabetic patients. Furthermore, when expressed relative to oxidative enzyme content, obese-diabetic patients tended to have higher muscle glycogen content, although differences were not statistically significant. Together these studies indicate that glycogen synthesis may be impaired early in the onset of skeletal muscle insulin resistance, and that the capacity of skeletal muscle to utilize fuel may be an important consideration.

#### 2.1.3. Mitochondrial Dysfunction

The disruption of normal mitochondrial biology can occur with insulin resistance long before the development of T2DM. Morino et al. [27] found that insulin-resistant offspring of patients with T2DM displayed a 60% lower insulin-mediated skeletal muscle glucose uptake and a 38% lower muscle mitochondrial density than healthy controls. Others have also reported differences in mitochondrial ultra-structure and size in T2DM [28, 29].

The oxidative capacity of skeletal muscle mitochondria may also be impaired in T2DM. Kelley et al. [28] found significantly reduced mitochondrial electron transport in the skeletal muscle taken from the vastus lateralis of participants with T2DM as compared to lean, healthy controls. Interestingly, Larsen et al. [30] found attenuated mitochondrial respiration in vastus lateralis muscle samples but not in mitochondria from the deltoid muscle from people with T2DM. Furthermore, using muscle biopsy samples, Ritov et al. [31] found that participants with T2DM (as compared to lean, healthy controls) had similar or higher production of reducing equivalents (NADH) from the Krebs Cycle and *β*-oxidation. Taken together, these studies indicate an impaired oxidative capacity of skeletal muscle in T2DM.

The delivery of normal or excessive amounts of reducing equivalents to a poorly functioning electron transport chain (described above) describes an environment that would favor a “backlog” in energy metabolism. This backlog in metabolism is associated with the accumulation of specific intermediates of lipid metabolism including fatty acyl-CoA, ceramides, and diacylglycerols. All of these intermediates correlate with insulin resistance and directly alter the insulin signaling cascade described previously [32].

#### 2.1.4. Accumulation of Lipid

Excessive accumulation of lipid within muscle has been shown to contribute to insulin resistance in both human and animal models [4, 5]. The accumulation is potentially due to the combination of the impaired ability of mitochondrial function (whether due to diabetic etiology, sedentary behavior, or both) and increased uptake of lipid into skeletal muscle under postprandial conditions. Kelley and Simoneau [33] found that in the postprandial state, fatty acid uptake was higher in patients with T2DM, where fat oxidation was reduced (supporting the progressive increase in muscle-lipid storage).

Lipid accumulation can occur both around muscle cells (extramyocellular lipids are stored within adipocytes within muscle tissue) and within muscle fibers (intramuscular triglycerides). According to Szcepaniak et al., intramuscular triglycerides are increased in obesity, and store quantity is positively related to severity of insulin resistance. Furthermore, increased intramuscular triglycerides are present in first-degree relatives of patients with T2DM, indicating that these stores may be an early indication of insulin resistance [34].

Histochemical techniques have revealed that the volume of lipid droplets in skeletal muscle is increased in T2DM. Approximately 1.5% of myocyte volume is occupied by lipid droplets in healthy controls, and 3-4% of muscle volume is occupied by lipid droplets in patients with T2DM [4]. Furthermore, the distribution of lipid droplets differs between healthy controls and patients with T2DM, where the latter display lipid droplets more centrally located within the muscle fiber [35]. The differing location of lipid droplets may affect availability of the substrate for oxidation [28].

In addition to disrupting muscle metabolism, the accumulation of lipid may have important functional implications. Examining a cohort of 2,979 participants from the Health ABC study, Visser et al. [36] found that reduced muscle attenuation (assessed via computed tomography; indicator of fat infiltration into the muscle) was associated with poorer lower extremity performance independent of muscle area. Age-related fatty-infiltration of skeletal muscle is also associated with the incidence of mobility disability [37].

### 2.2. Dysfunction in Bone in T2DM

#### 2.2.1. Osteoporosis and Reduced Bone Quality

Several studies suggest that diabetes negatively impacts skeletal health and may increase one's risk of skeletal dysfunction [38, 39]. Osteoporosis is the most common metabolic skeletal disease in the United States and is characterized by low bone mineral density (BMD) resulting in an increased risk for fractures [40]. The National Osteoporosis Foundation has indicated that approximately 10 million Americans have osteoporosis (T-scores ≤−2.5) and an additional 34 million are osteopenic (T-scores between −1 and −2.5) [8]. With more than 2 million osteoporotic fractures in 2005, osteoporosis-related medical expenses reached $19 billion and are expected to grow to $25.3 billion by 2025. Fracture rates are even higher for diabetic populations [41, 42]. The risk of fractures varies by body site, with fracture risk being more than double in certain sites and an overall fracture risk 29% higher in diabetics compared to nondiabetics [7]. The drastic increase in fracture risk strongly links compromised bone health with diabetes. Findings in animal models indicate that fracture risk may be related to a reduced ability of diabetic bone to withstand load and bending. Reddy et al. [39] examined the femur and tibia in experimentally induced diabetic rats and found that diabetes reduced the femur and tibia mean maximum load 37 and 30%, deformation at maximum load 25 and 30%, and energy absorbed 27 and 23% compared to controls. Additionally, bending stiffness increased in the diabetic rat bones: femur 57% and tibia 38%. Taken together, these studies indicate a reduced bone quality in T2DM. There are several potential causes for reduced bone quality in T2DM including bone cell formation, advanced glycation end products, and lipid accumulation.

#### 2.2.2. Bone Cell Formation

Healthy bone tissue undergoes a constant state of remodeling, a coupled process involving resorption and formation of bone through the activation of osteoclasts, osteoblasts, and osteocytes [43, 44]. Ideally, the osteoclast resorption of bone is closely coupled with osteoblast bone formation in adults to maintain healthy bone structure [45]. T2DM, however, may interfere with or alter these processes resulting in compromised bone density and quality, elevating one's risk for osteoporosis and fractures [8, 46]. Mesenchymal stem cells (MSCs), located in the bone marrow, are one form of multipotent cells having the potential to give rise to a variety of cell types including bone, fat, cartilage, and marrow [47]. The differentiation of MSC to either osteoblasts or adipocytes is mediated by a variety of molecular, biochemical, and physical stimuli [48]. Wang et al. [9] utilized human osteoblast-like MG-63 cells to determine the impact of high glucose levels on MSC. The differentiation of MSC to either osteoblasts or adipocytes is regulated in part by runt-related transcription factor 2 (Runx2) and peroxisome proliferator-activated receptor *γ* (PPAR*γ*). Runx2 favors osteoblast formation while PPAR*γ* favors adipocyte formation. Elevated glucose not only decreased the development and maturation of MG-63 cells by 30–40% but also reduced levels of osteogenic markers Runx2, collagen I, osteonectin, and osteocalcin [9]. Though these findings have provided a potential contributor to reduced bone quality in T2DM, future studies should examine whether hyperglycemia in T2DM impacts bone formation.

#### 2.2.3. Advanced Glycation End-products (AGEs)

AGE may play a role in both bone cell formation and other factors related to bone quality. AGEs result from the nonenzymatic reaction of reducing sugars (such as glucose and fructose) with proteins or lipids—a process called glycation. Continued glycation of these products leads to molecular rearrangements and the ultimate production of AGE [49]. AGE can produce reactive oxygen species, bind to specific cell surface receptors, and form cross-links. Since advanced glycation can take weeks to occur endogenously, the primary effectors are long-lived proteins, including connective tissue such as collagen [50]. Alikhani et al. [51] determined that AGEs induce an apoptotic effect on osteoblast cells diminishing bone formation which was found to be largely mediated through AGE receptors. Saito et al. [52] investigated diabetic induced alterations in collagen cross-links utilizing rats. The two general divisions of collagen cross-links include enzymatic cross-links, which have a positive effect on bone function, and nonenzymatic cross-links, which impair bone function. Saito et al. [52] reported that an elevation in glycation-induced pentosidine (Pen), a common marker for cross-linking AGE present in bone, impaired the mechanical properties of bone in spontaneously diabetic rats. The level of Pen and the Pen to total enzymatic cross-links (P/TECLs) ratio had a significant relationship with the mechanical properties of bone. Both Pen and Pen/TECL ratio were associated with a decrease in the energy absorption, stiffness, elasticity, and maximum load.

These findings support the idea that AGE and collagen may be of particular importance in T2DM bone. Collagen serves to enhance the toughness and tensile strength of bone due to its capacity to absorb energy [52, 53]. Non-enzymatic collagen cross-linking has been shown to impair the mechanical properties of bone [51, 54] thereby making it more susceptible to fractures. Additionally, AGE have been shown to induced reductions in osteoblasts which can also compromise bone health [55]. Thus, it has been speculated that reducing AGE could help maintain bone quality and reduce the diabetic associated fracture risk [56]. It is important to note that assessments of BMD often do not detect changes in bone quality, warranting alternative assessments of bone health in diabetic populations.

#### 2.2.4. Lipid Accumulation

Diabetes may also lead to an increase in bone adiposity [8] thereby reducing bone quality. Utilizing streptozotocin-induced diabetic mice, Botolin et al. [57] reported an increase in marrow adiposity mediated by an elevation in PPAR *γ*2, resistin, and adipocyte fatty acid binding protein. Additionally, lipid-dense adipocytes were noted in the tibia of the diabetic mice. Whether diabetes induced differentiation of mesenchymal pluripotent cells to adipocytes, or if accumulation of lipid occurred in existing adipocytes making them more visible, is unclear. The accumulation of lipid in bone reportedly expands the marrow cavity decreasing the cortical envelope [8].

The increase in adipogenic markers found under high glucose conditions may be one factor contributing to the adiposity of bone with diabetes. Wang et al. [9] determined that high glucose conditions suppressed osteogenic differentiation of MG-63 cells and elevated adipogenic differentiation. The increased adiposity was attributed to an elevation of adipogenic markers: PPAR*γ*, adipsin, resistin, and aP2 as the result of high glucose levels. Thus, hyperglycemia associated with diabetes may alter the normal regulation of bone and lead to increased adiposity resulting in compromised bone quality.

## 3. Role of Resistance Exercise in Treating Musculoskeletal Dysfunction

The importance of exercise for patients with T2DM is emphasized by major organizations including the American College of Sports Medicine and the American Diabetes Association. Exercise is considered a cornerstone in the therapeutic intervention for patients with T2DM, and the importance of exercise is underscored by the costs and side effects that accompany pharmacological intervention. Two major modes of exercise are aerobic exercise and resistance exercise. Aerobic exercise involves exercises performed with large muscle groups over extended periods involving hundreds of repetitions and limited by the delivery of oxygen to the working muscles. Resistance exercise (also referred to as resistance training) involves the movement of high loads using resistance from either machines or weights for a smaller number of repetitions [58, 59].

Although cardiovascular exercise is often encouraged as a nonpharmacological method to manage T2DM, complications associated with diabetes often hinder cardiovascular capabilities [60, 61] and only 39% of diabetics meet the American Diabetes Association recommendations of 150 min of moderate-intensity or 90 min of vigorous-intensity cardiovascular exercise per week [55]. More recently, resistance training has been found to be effective for managing T2DM and may provide the additional benefit of preventing or limiting musculoskeletal dysfunction associated with T2DM [54, 60, 62, 63].

The majority of clinical trials that have examined the impact of exercise on T2DM and associated comorbidities have employed aerobic modes of exercise. Wang and Jin [58] provided an excellent systematic review about the adaptations to exercise training in skeletal muscle in T2DM; 78% of the studies that met the inclusion criteria prescribed aerobic training. Irvine and Taylor [11] conducted a systematic review of resistance exercise on glycemic control in T2DM and found nine trials that met inclusion criteria. Resistance training appears to have an important impact on T2DM; resistance exercise has been shown to improve HbA1C significantly more than performing no exercise at all and similarly to aerobic exercise in patients with T2DM. Furthermore, resistance exercise can cause significantly better improvements in strength when compared to aerobic training [11]. 

The few trials that have included only resistance exercise have focused largely on systemic physiological and functional responses more so than the direct impact of the training on skeletal muscle and bone. Since resistance training has a meaningful positive effect clinically on people with T2DM, and given the substantial dysfunction present in skeletal muscle and bone of people with T2DM, it is important to explore the impact of resistance exercise on this dysfunction. Although some direct evidence about the impact of resistance exercise exists with respect to the dysfunction in skeletal muscle with T2DM, there is little to no direct evidence about the impact of resistance exercise on the dysfunction in bone with T2DM. Therefore, the purpose of this section of the review is (1) to examine how resistance exercise impacts the aforementioned dysfunctional aspects of skeletal muscle and (2) to hypothesize how resistance exercise may impact the dysfunction found in bone in T2DM. Through these hypotheses, we hope to highlight areas in need of additional research with respect to bone dysfunction in T2DM. This review does not include trials where aerobic exercise and resistance exercise were combined or where aerobic exercise alone was employed.  Table 1 provides a summary of the resistance training protocol in trials with patients who had T2DM.

### 3.1. Insulin-Independent Glucose Uptake in Exercising Muscle

In addition to insulin-mediated glucose uptake, glucose can also be taken up into muscle through insulin-independent mechanisms (contraction-mediated uptake), which predominate during exercise. It appears that there are distinct contraction and insulin-responsive GLUT4 pools in skeletal muscle [73, 74], and contraction does not stimulate the cascade of events in insulin signaling [75]. Two potential mechanisms of insulin-independent glucose uptake have been described, one mediated via intracellular calcium levels, and the other dependent upon 5′AMP-activated protein kinase (AMPK) [76]. It appears that the extent to which muscle glucose uptake increases during exercise is due to exercise intensity [65], supporting the idea that both of these proposed pathways may work in concert. Given the normal reduction in serum insulin during exercise, these pathways are a critical means of fuel delivery.

The insulin-independent uptake of blood glucose appears to function normally in people with T2DM even when insulin action is impaired [77]. Musi et al. [68] demonstrated that a single bout of aerobic exercise (45 minutes at 70% of maximum work load) significantly reduced blood glucose, and increased AMPK alpha2 activity to a similar degree as compared to health controls. Since this pathway appears to work normally in T2DM, exercise provides an excellent opportunity to manage blood glucose. As such, exercise can be an important means to improve glycemia in T2DM via these mechanisms in addition to any impact exercise may directly have on areas of dysfunction in skeletal muscle.

### 3.2. Resistance Exercise Effects on Dysfunction in Muscle Identified in Section 2

#### 3.2.1. Insulin Resistance

Whole-body insulin resistance, as estimated by the homeostasis model assessment (HOMA-IR), has been shown to improve by ~25% after 16 weeks of whole-body strength training three times per week in older Hispanic adults with T2DM [54]. However, given that HOMA-IR is calculated using fasting values of glucose and insulin, this method is more indicative of hepatic insulin sensitivity [3]. Dunstan et al. [67] randomized patients with T2DM to circuit weight training program three times per week for eight weeks or to a nonexercising control group. An oral glucose tolerance test (OGTT) was performed at baseline and week eight. Both glucose and insulin area under the curve decreased significantly in the intervention group from baseline to week eight, and reductions in insulin area under the curve remained significant after adjusting for changes in body mass. In contrast, Baldi and Snowling [64] found that a 10-week resistance training program performed three times per week did not change 2-hour glucose or insulin but did lead to reductions in HbA1c and fasting insulin in patients with T2DM. Ibañez et al. [12] assigned nine older men with T2DM to a progressive resistance training program twice per week for 16 weeks. Insulin sensitivity, as measured using the frequently sampled intravenous glucose tolerance test, improved by 46.3%. Of note, these participants had no significant change in body weight and increased energy intake by 15% over the course of the study.

The euglycemic insulin clamp technique is considered the gold standard for the *in vivo* measurement of insulin action [78]. Insulin sensitivity measured using this technique is primarily a reflection of skeletal muscle insulin sensitivity as described by Ferrannini et al. [79]. As such, studies employing this method to assess insulin sensitivity are best suited to examine the effects of resistance training on skeletal muscle insulin sensitivity. Ishii et al. [69] assigned 17 patients with T2DM to either a resistance exercise group (2 sets of 9 exercises 5 times per week) or a nonexercising control group for 4–6 weeks. The intervention group experienced a 48% increase in glucose disposal rate (as assessed using euglycemic insulin clamp), indicating improved skeletal muscle insulin sensitivity. Interestingly, VO2peak (a measurement of aerobic capacity) was unchanged. Using similar techniques, Holten et al. [70] found increased muscle glucose uptake after single-leg strength training 30 minutes per day, three times per week for six weeks. These studies indicate that strength training increases skeletal muscle insulin sensitivity; notably, these changes occurred after only short-term training (4–6 weeks). Furthermore, the improvements seen in insulin sensitivity following resistance training are of a similar magnitude seen following aerobic training [15, 80].

#### 3.2.2. Impaired Glycogen Synthesis

It appears that resistance exercise may be an effective means for improving glycogen and glycogen synthase in muscle. Holten et al. reported significant increases in glycogen content, glycogen synthase protein content, and glycogen synthase activity after six weeks of resistance exercise in T2DM patients [70]. Similarly, Castaneda et al. found that 16 weeks of resistance exercise three times per week increased muscle glycogen by ~32%; interestingly, a control group who performed no exercise experienced a significant reduction in muscle glycogen [66]. However, not all studies have shown improvements in muscle glycogen levels following a resistance exercise program. Praet et al. [72] trained patients with T2DM using a resistance exercise program three times per week for 10 weeks and found no significant change in muscle glycogen content, despite significant reductions in fasting glucose and exogenous insulin requirements.

#### 3.2.3. Mitochondrial Dysfunction

Although changes in skeletal muscle mitochondrial morphology and function after aerobic training have been studied extensively, very few studies have examined the impact of resistance training on mitochondrial quantity and size in T2DM. However, resistance exercise has been shown to increase mitochondria in other populations. Balakrishnan et al. [81] reported that a resistance exercise program completed three times per week for 12 weeks significantly increased skeletal muscle mitochondrial content in older adults with chronic kidney disease. Resistance exercise has also been shown to improve mitochondrial function in older adults [82].

The response of skeletal muscle oxidative activity to resistance training in patients with T2DM has been examined, but in very few studies. Holten et al. [70] found similar levels of oxidative enzymes (citrate synthase and hydroxyl-acyl-CoA dehydrogenase) in patients with T2DM and controls previous to a 6-week resistance training intervention and reported no significant changes in these enzymes following the training program. In agreement, Praet et al. [72] reported that resistance training performed three times per week for 10 weeks resulted in no significant change in skeletal muscle succinate dehydrogenase activity.

The effects of resistance exercise on mitochondrial morphology and function in healthy adults have been more widely examined. Resistance exercise in healthy, untrained males has been shown to reduce the relative skeletal muscle volume of mitochondria and density [83, 84]. However, in both of these studies, muscle fiber cross-sectional area increased significantly, leading to the conclusion (in both cases) that resistance training results in a “dilution of the mitochondrial volume” in skeletal muscle. Interestingly, and similarly to patients with T2DM, the oxidative potential in sedentary healthy adults has also been largely found unresponsive to chronic resistance training [85–90]; however, some reports of both increases [91–93] and decreases [94] in this population also exist.

#### 3.2.4. Accumulation of Lipid

Very few interventions exist to elucidate the effects of a resistance training program on muscle lipid content in people with T2DM. After 10 weeks of progressive resistance exercise in patients with T2DM, Praet et al. [72] reported no change in the lipid content of Type I, Type IIa, or Type IIx muscle fibers, and the lipid content in Type I fibers was greater than that in Type IIa and Type IIx fibers at both baseline and postintervention analyses. Ku et al. [71] examined the effects of strength training (using elastic bands) at 40–50% of maximal strength five times per week for 12 weeks in 44 Korean women with T2DM. Subcutaneous, subfascial, and intramuscular adipose were measured in the mid-thigh prior to and following the resistance training program. No significant changes in any of these adipose depots were detected after the training program. There was also an aerobic-trained group in this study who also had no changes in muscle lipid content.

The effects of resistance exercise alone on muscle lipid content in other populations have also been scarcely reported. Mueller et al. [95] assigned elderly men and women to either an eccentric-based or conventional strength training program twice per week for 12 weeks. Intramyocellular lipid content decreased in the eccentric-based program, but not in the conventional program—possibly indicating that the tempo of resistance training may play a role in outcomes. Given the numerous benefits of resistance exercise alone in T2DM, future studies are needed to explore the effects of this type of training on muscle lipid content.

### 3.3. Hypothesized Effects of Resistance Training on Dysfunction in Bone

#### 3.3.1. Weight Bearing, Resistance Training, and Bone

Bone functions to withstand internal and external forces and is able to adapt to enhance its ability to endure future loading conditions. The mechanical loading of bone via weight-bearing activities induces temporary bone deformation [96] known as strain [97]. Weight-bearing exercise, including resistance training, can provide the appropriate strain necessary to the activate bone formation process through mechanotransduction. Mechanotransduction involves “the conversion of a biophysical force into a cellular response” [97]. The strain associated with resistance training results in mechanical stretching of osteocytes and bone lining cells and hydrostatic pressure gradients causing the movement of interstitial fluid within the canaliculae of bone. Fluid movement creates shear stress on the osteocytes, bone lining cells, and osteoblasts. The stretching and shear stress placed on these cells during this process leads to the formation and activation of osteoblasts to form new bone in the affected area. Subsequently over time, this process can lead to increases in BMD. The potential influence that weight-bearing activities have on bone can be affected by several factors including strain magnitude, strain rate, strain frequency, and strain variability [98, 99].

Weight-bearing activities can induce varying degrees of strain [100]. A minimal effective strain (MES), roughly 1,500 *μ* strain or greater, is necessary to stimulate bone formation. When strain magnitudes are not of a sufficient threshold, bone resorption can outweigh bone formation resulting in a loss of bone [97]. Thus, presence or absence of weight-bearing activities can impact bone health dramatically. Under extended conditions of unloading, such as spaceflight or bed rest, the lack of mechanical loading produces insufficient strain magnitudes, which can result in excessive bone resorption and decreases in BMD [101], thereby justifying the need for regular weight-bearing activity to maintain BMD and overall bone health. The magnitude of strain necessary to reach MES can vary dramatically when considering variables such as strain rate, frequency, and variability [96]. Higher strain rates magnify the speed of fluid movement enhancing shear stress creating a greater osteogenic stimulus even at lower strain magnitudes [96]. Greater strain frequency reduces the need for high strain magnitudes to induce an osteogenic response [100]; however, bone appears to lose sensitivity to loading after a certain number of loading cycles and continued loading may not provide any additional benefit [98]. Rest inserted between loading has been shown to optimize the sensitivity of bone to future loading, maximizing the osteogenic response [99]. Varying the direction of the strain creates differing force vectors thus enhancing stimulation and the formation of new bone in a greater diversity of locations.

Weight-bearing exercise is well recognized for its influence on the bone remodeling process enhancing BMD [96, 102]. This benefit is often observed through comparisons of sports with diverse weight-bearing parameters and comparison made between athletes and nonathletes [102, 103]. Athletes in general tend to have higher BMD than nonathletes and BMD tends to be greatest in athletes who participate in sports that generate greater ground reaction forces [102]. Resistance training is a form of weight-bearing activity that has osteogenic potential. Resistance training has been found to alter biomarkers for bone formation and bone resorption [104]. Fujimura et al. [104] reported an increase in osteogenic markers following 4 months of resistance training, but no changes in BMD were observed. It was speculated that the changes in biomarkers preceded any detectable changes in BMD.

Longer duration resistance training studies have however reported improvements in BMD. Almstedt et al. [105] found a 2.7 to 7.7% increase in the BMD of male participants following a 24-week resistance-training program but no significant changes in the BMD of female participants. Nichols et al. [106] reported significant increases in femoral neck BMD in resistance trained females compared to nontrained controls. Following a 9-month resistance training program Pruitt et al. [107] found not only increases in BMD in resistance-trained early postmenopausal female subjects but also a decline in BMD in nontrained subjects. While not all studies report increases in BMD with resistance training, resistance training may help maintain existing bone mass or slow the loss of BMD which may be just as beneficial with respect to preventing osteoporosis.

A substantial amount of research has identified the benefits of weight-bearing exercise such as resistance training, on bone in healthy populations [102–107]. Resistance training has been found to have variable but often positive benefits on BMD [108]. Limited research however exists examining the impact of resistance training on diabetic skeletal dysfunction. Diabetes was found to be a common exclusionary criterion for resistance training and bone research. One study was discovered that utilized diabetic subjects. Daly et al. [109] determined that resistance training was beneficial at helping maintain BMD in diabetic participants subjected to a moderate weight loss program compared to a loss in BMD seen in the weight loss only group. In diabetic populations however, utilization of BMD as a mark of bone health is not always appropriate because it does not equate to fracture risk [43]. Thus, examining the impact of resistance training on the factors associated with reduced bone quality in diabetic populations may help shed light on the potential benefits of resistance training in diabetic populations. This is an important area of research that warrants exploration. However, until such research is conducted utilizing diabetic subjects, we can only hypothesize about the impact resistance training has on bone quality by examining its impact on bone in healthy subjects. The following studies provide evidence regarding the benefits of resistance training to factors that have been reported to compromise bone quality in diabetics. So, although these studies do not utilize diabetic subjects, they provide insight regarding the potential benefits resistance training may serve to correct diabetic abnormalities that impact bone.

#### 3.3.2. Advanced Glycation Endproducts

Hyperglycemia and AGE negatively impact bone in a variety of ways. Hyperglycemia reduces osteogenic cellular differentiation and osteogenic markers [9]. No research was found examining the effect of resistance training on osteoblast differentiation or osteogenic markers in diabetics; however, resistance training has been found to promote osteogenic cellular differentiation and osteogenic markers utilizing animal models and nondiabetic subjects, respectively. Menuki et al. [110] found an increase in osteoblast differentiation in bone marrow cells from the tibia and femur of mice following 28 days of stair climbing exercise. Humans with a history of resistance training also show increased osteogenic activity. Fujimura et al. [104] reported an increase in biomarkers for osteogenesis, including osteocalcin, and a decrease in urinary deoxypyridinoline (DPYR), a marker of bone resorption, in resistance trained subjects. Osteocalcin levels increased significantly from baseline each month during the four months of training while DPYR decreased during the first three months and then returned back toward baseline. BMD of the resistance-trained subjects did not increase following the four-month time period. The researchers concluded that increases in the biomarkers for osteogenesis may precede any noticeable changes in BMD. The positive changes in osteoblast differentiation and osteogenic biomarkers via resistance training have the potential to reduce the negative effect of diabetes on these specific parameter that impact bone quality.

AGEs have a deleterious effect on bone by reducing osteoblast cells [51] and increasing nonenzymatic collagen cross-links impairing bone quality [111]. No literature was found examining the potential alterations to the influence of AGE on bone with any form of exercise, including resistance training. Limited research appears to exist examining the impact of exercise in general on AGE. Boor et al. [112] reported a decrease in AGE in obese Zucker rats following 10-weeks of moderate aerobic exercise. Magalhães et al. [113] therefore have speculated that exercise may aid in the reduction and accumulation of AGE. Since resistance training has been identified as an effective form of exercise for managing T2DM [54, 60, 62, 63], the potential exists for such training to impact AGE; however, research is needed to fully elucidate such a connection.

#### 3.3.3. Lipid Accumulation

Compromised bone quality with diabetes is due in part to an increase in bone adiposity [8]. Accelerated adipocyte differentiation coupled with a decrease in osteoblast differentiation has been found under high glucose conditions in animal models [9, 57]. No research exists to date examining the impact of resistance training on the alterations to lipid accumulation in diabetic bone. Menuki et al. [110] did however determine that mechanical loading induced alterations to the cellular differentiation of bone marrow cells utilizing an animal model. Menuki et al. [110] examined bone samples taken from the tibias and femurs of 70 mice following either 4, 7, or 28 days of stair climbing. Adipocyte differentiation was significantly lower in the 28-day stair climbing mice compared to control group mice, indicating that the mechanical loading had a positive effect on decreasing bone adiposity. MSCs have also been found to favor osteoblast differentiation when exposed to a mechanical strain [114]. Sen et al. induced a 2% mechanical strain at 10 cycles per minute for 3600 cycles total on cultured MSC. The researchers determined that the mechanical strain reduced PPAR*γ* availability, thereby limiting bone adiposity and favoring osteogenesis, indicating that the functional loading of the skeleton through exercise is important to bone mass and morphology. Zayzafoon et al. [47] provide further support regarding the necessity of mechanical loading to the maintenance of bone quality. Zayzafoon et al. created a microgravity (MG) environment to investigate the impact of unloading, as occurred with space flight, on MSC differentiation. The cells underwent 7 days of MG. The researchers determined that the 7 days of MG totally suppressed osteoblast formation mediated by a Runx2 inhibition, indicating the need for mechanical stimulation to maintain MSC differentiation in favor of bone formation. Although these studies did not involve diabetic conditions, we hypothesize that these lend support to the potential for resistance training to alter cellular differentiation in favor of improving bone quality in diabetics.

## 4. Conclusions

### 4.1. Summary

Diabetes effects more than 346 million people worldwide [2] and has substantial impact on the development of a wide array of comorbidities, subsequently decreasing quality of life, and life-expectancy [115]. T2DM has been identified as a contributing factor to musculoskeletal dysfunction. In muscle tissue, dysfunction occurs with insulin sensitivity [3], mitochondrial function [6], and an accumulation of triglycerides [4, 5]. Some [66] but not all [18] studies indicate that glycogen synthesis is improved by resistance training in T2DM. Both mitochondrial function and accumulation of lipid have only been examined to a limited capacity in T2DM, and the limited research indicates a minimal impact of resistance training on these areas of dysfunction.

Skeletal dysfunction consists of altered bone quality mediated by hyperglycemia [9], AGE [51, 52], and lipid accumulation [8, 9, 57]. Despite T2DM potentially increasing BMD, evidence of elevated fracture risk indicates poor bone quality. Bone quality is compromised as a result of many factors. Diabetes appears to increase nonenzymatic collagen cross-linking [51, 54] and decrease osteogenic activity by reducing osteoblast cell growth and differentiation [9], while promoting adipocyte differentiation and subsequently increasing the adiposity of bone [8, 57]. Given the limited amount of research done with respect to resistance training in T2DM, while taking into consideration the related findings presented previously, we have hypothesized that (1) the impact caused by resistance training may improve bone quality, and (2) the improvements in glycemic control resulting from resistance training may cause a positive shift in bone cell formation (and a shift away from lipid formation).

Pharmacological methods are often employed to help manage T2DM; however some T2DM pharmaceuticals compromise the health of other body systems. For example, thiazolidinedione has been implicated to suppress bone formation and increase the risk for fractures [116, 117]. The consequences associated with pharmacological diabetes management further warrant the need for nonpharmacological interventions. Although lifestyle interventions, such as exercise, may have an associated financial cost, as do pharmaceuticals, exercise also has expansive health and quality of life benefits. As such, we acknowledge that a comprehensive lifestyle intervention will impose some financial demand on the patient, and that the cost-effectiveness of lifestyle-related therapy in comparison to pharmacological therapy needs further investigation.

It is also important to recognize that the majority of the studies reviewed employed supervised resistance training programs. As such, the results presented are only applicable clinically when resistance training is supervised, particularly in light of findings by Dunstan et al. [118] that demonstrated a reduced adherence and training volume and intensity when patients with T2DM switched from supervised strength training to home-based strength training.

### 4.2. Suggestions for Future Research

In an effort to more clearly understand the potential benefits of resistance training on musculoskeletal dysfunction with T2DM, future research should focus on increasing the use of T2DM subjects in resistance training studies. In addition to better understanding volume/intensity thresholds (for practical reasons), the mechanistic examination of the unique effects of resistance training in this population is important.

1. There are several devastating health-related consequences of diabetes including osteoporosis which can dramatically alter one's quality of life and have medical costs. Research is strongly needed to help fully understand diabetic skeletal dysfunction and to explore the potential benefits of resistance training to combat the dysfunction.
2. As specifically identified by this review, a mechanistic understanding about the effects of resistance training in T2DM on the following is needed: mitochondrial quantity and size, and lipid accumulation in muscle, as well as AGE, osteoblast differentiation, and lipid accumulation in bone.

## Figures and Tables

**Table 1:** Summary of resistance training protocol in trials with patients who have type II diabetes.

Study [reference no.]	Duration	Training frequency	Number of Exercises	Sets × Reps	Intensity (%1RM)	Compliance	Training effect	Body weight
Baldi and Snowling [64]	10 wks	3x/wk	10	Wk1: 1 × 12 Wk2–10: 2 × 12	NR	89.6%	Upper and Lower Body Strength ↑8–37%	+1.7 kg
Rose and Richter [65]	Paper reporting from same cohort presented in Castaneda et al. (Below)							
Castaneda et al. [66]	16 wks	3x/wk	5	3 × 8	60–80%	90 ± 10%	Upper body Strength ↑36% Lower body Strength ↑51%	+0.2 kg
Dunstan et al. [67]	8 wks	3x/wk	9	Wk1-2: 2 × 10–15 Wk2–8: 3 × 10–15	50–55%	NR	Strength ↑ for all exercises (↑range: 15–43%)	−0.4 kg
Musi et al. [68]	24 wks	3x/wk	9	3 × 8–10	Wk1-2: 50–60% Wk3–24: 75–85%	88%	Upper body Strength ↑43% Lower Body Strength ↑33%	−2.5 kg
Ibañez et al. [12]	16 wks	2x/wk	7-8	Wk1–8: 3-4 × 10–15 Wk9–16: 3–5 × 5-6	Wk1–8: 50–70% Wk9–16: 70–80%	99.3%	Upper Body Strength ↑18.2% Lower Body Strength ↑17.1%	n/c
Ishii et al. [69]	4–6 wks	5x/wk	9	2 × 10 Upper Body 2 × 20 Lower Body	40–50%	100%	Lower Body Strength ↑16%	BMI ↓0.6 kg/m^2^
Holten et al. [70]	6 wks	3x/wk	3*	Wk1-2: 3 × 10 Wk3–6: 4 × 8–12	50% 70–80%	100%	Lower Body Strength ↑42–75%	n/c
Ku et al. [71]	12 wks	5x/wk	10	3 × 15–20	40–50%	NR	Upper body Strength ↑12% Lower body Strength ↑11%	−1.1 kg
Misra et al. [13]	12 wks	3x/wk	6	2 × 10	NR	100%	NR	n/c
Praet et al. [72]	10 wks	3x/wk	5	2 × 10	50–60%	83%	Upper body Strength ↑16% Lower body Strength ↑18%	−0.1 kg

RM: repetition maximum; training frequency reported as times per week (x/wk); sets and repetitions reported as number of sets by the number of repetitions (sets × reps); wks: weeks; wk: week; kg: kilograms; BMI: body mass index; m^2^: meters squared; NR: not reported; n/c = no change; *strength training was performed on one leg only throughout the study; the other leg remained sedentary.
